# Is direct bodyguard manipulation a parasitoid-induced stress sleep? A new perspective

**DOI:** 10.1098/rsbl.2022.0280

**Published:** 2022-11-30

**Authors:** Prabitha Mohan, Palatty Allesh Sinu

**Affiliations:** ^1^ Department of Zoology, Central University of Kerala, Kasaragod, Kerala, India; ^2^ Zoological Survey of India, Chennai, Tamilnadu, India

**Keywords:** host manipulation, parasitoids, bodyguard behaviour, stress-induced sleep

## Abstract

Bodyguard manipulation is a behavioural manipulation in which the host's behaviour is altered to protect the inducer's offspring from imminent biotic threats. The behaviour of a post-parasitoid-egressed host resembles a quiescence state with a characteristic reduction in motor activities like feeding, locomotion, respiration, and metabolic rate. Yet, they respond aggressively through a defensive response when disturbed, which ensures better fitness for the parasitoid's offspring. The behavioural changes in the parasitized host appear after the parasitoid egression. Several hypotheses have been proposed to elucidate how the parasitized host's behaviour is manipulated for the fitness benefits of the inducers, but the exact mechanism is still unknown. We review evidence to explain the behavioural changes and their mechanism in the parasitized hosts. The evidence suggests that parasitoid pre-pupal egression may drive the host to stress-induced sleep. The elevated octopamine concentration also reflects the stress response in the host. Given the theoretical links between the behavioural and the physiological changes in the post-parasitoid-egressed host and stress-induced sleep of other invertebrates, we suggest that behavioural studies combined with functional genomics, proteomics, and histological analyses might give a better understanding of bodyguard manipulation.

## 1. Introduction

Parasites have evolved multiple host manipulative strategies for their survival and transmission [1–3]. Parasitoids are parasitic insects that kill their hosts—predominantly the immature stages of other insects and spiders—during their development [4]. Like other parasites, some parasitoids have evolved a host manipulative strategy called bodyguard manipulation [4–6]. The bodyguard-manipulated host protects the parasitoid juvenile from biotic threats like predators and hyperparasitoids [7]. For example, *Pieris brassicae* (Pieridae)*,* parasitized by braconid parasitoid *Cotesia glomerata,* guards the parasitoid pupa by responding aggressively to approaching predators [8].

All the parasitoids which induce bodyguard manipulation are koinobionts—the parasitoids that do not paralyse the host during parasitization. So, their host will continue to develop even after oviposition. Unlike predators, which can feed on many prey during their development, parasitoids have only limited food sources from a single host [9]. The eggs of koinobiont parasitoids are yolk-deficient, and they absorb nutrients from the host haemolymph [10]. For optimal resource utilization, koinobiont parasitoid juveniles follow either of two strategies. First, parasitoids feed on most host tissues before the pupation, which is considered plesiomorphic. The parasitoids following this strategy kill their host when they pupate. Second, the parasitoids feed only on the host's haemolymph and will not damage any major host tissues, this is considered an apomorphic strategy. The parasitoids using this strategy emerge out of the host cuticle by perforating the sides of the host body and pupate near the live host. The host remains alive for a few days with the pupa of the parasitoid and eventually dies owing to starvation [9].

## 2. Bodyguard manipulation: types and strategies

Bodyguard manipulation is mainly induced by parasitoid wasps in order to protect the vulnerable pupal stage from their natural enemies. It can be of two types based on the resource utilization strategy of the manipulating parasitoids—direct bodyguard manipulation and indirect bodyguard manipulation [11]. In direct bodyguard manipulation, the host will be alive even after the parasitoid pupation. The live host guards the vulnerable pre-pupal stage of parasitoids against the approaching natural enemies—predominantly hyperparasitoids and insect predators. The manipulated host also stops its feeding and locomotion only after the parasitoid egression. The parasitoid juveniles perforate the host cuticle and emerge from the host body with the help of their mandibles and terminal appendages [12]. The cessation of feeding at this stage also protects the parasitoid pre-pupa from the attack of the host itself [13].

Most case studies on direct manipulation are induced by the Microgastrinae subfamily of Braconid parasitoids. In Microgastrinae, juveniles of more recently evolved genus feed only on the host haemolymph. So their host will survive with minimal damage even after parasitoid egression compared to tissue-feeding parasitoids [14]. Following are some examples of direct bodyguard manipulation. A gregarious Microgastrinae parasitoid *Cotesia congregata* pupates on the dorsal and lateral cuticle of its live bodyguard host *Manduca sexta* (Sphingidae)*.* Experimental studies indicate that the survival rate of parasitoid cocoons increases to 80% when they are near the host rather than away from the host [9]. After the parasitoid emergence, the host stops feeding and locomotion [9]. Similarly, in the case of *P. brassicae,* parasitized by *Cot. glomerata,* the host remains alive near the parasitoid cocoon cluster and spins a web over it. The host larva also responds aggressively towards the approaching predators [15]. In another system, *Thyrinteina leucocerae* (Geometridae), parasitized by the parasitoid, *Glyptapanteles* sp., stops feeding and guards the parasitoid's pupa against predators through violent head swings [16]. *Microplitis mediator,* a solitary Microgastrinae parasitoid of *Mythimna separata* (Noctuidae), makes its parasitized host defend hyperparasitoids from their pupae [17,18]. Another species of *Microplitis, Microplitis pennatulae,* makes its parasitized host *Psalis pennatula* (Erebidae) guard its pupa against its hyperparasitoid, *Brachymeria lasus* (Chalcididae) (electronic supplementary material, video file S1)*. Microplitis pennatulae* pre-pupa emerges from the host body 11–14 days after parasitization. After parasitoid pre-pupal egression, the host exhibits behavioural changes. The parasitoid egressed host suspends feeding and locomotion and exhibits a defensive response to the thigmotactic stimulus. This response protects the parasitoid pupa from the hyperparasitoid *B. lasus* [19]. Under experimental conditions, when *Mi. pennatulae* pupae were exposed to the hyperparasitoids, all the unguarded pupa were readily hyperparasitized, while only 30% of the guarded pupae were hyperparasitized [19]. The hyperparasitoids that manage to approach the parasitoid pupae without disturbing the overlying host can succeed in parasitizing the parasitoid pupa (P. Mohan 2015, personal observation). *Dinocampus coccinellae,* a braconid parasitoid of the Helconoid subfamily, also induces direct bodyguard manipulation. Cocoons of *Di. coccinellae* suffer less predation when attended by the live host, *Coleomegilla maculata* (Coccinellidae)*,* than when not attended by the host. Parasitized *Col. maculata* also exhibits twitches at irregular intervals when disturbed [5].

In indirect manipulation, parasitoids follow the plesiomorphic strategy of resource utilization. So, the parasitoid juvenile feeds on the host tissues, kills the host during its emergence, and then goes for pupation. However, before the emergence, the parasitoid guides the host to a concealed location or prompts the host to create a protective shelter for the parasitoid juvenile to resist biotic threats. For instance, *Aphidius nigripes,* a braconid endoparasitoid of the subfamily Aphidiinae, pupates inside the eviscerated body of its host, *Macrosiphum euphorbiae* (Aphididae). The parasitoid larva induces its host to leave the colony and hide in a concealed location to escape hyperparasitization [20]. Some ichneumonid wasps prompt spider hosts to spin a cocoon web before killing the host [21–23].

Unlike indirect bodyguard manipulation, direct bodyguard-inducing parasitoids alter their host's behaviour after the parasitoid egression. Though these studies have recorded the behavioural changes involved in bodyguard manipulation, it is unclear who induces manipulation and what the mechanism used is.

## 3. How are behavioural changes induced in hosts?

Studies have postulated—neuronal, immunological, genomic/proteomic and symbiont-mediated mechanisms to explain host manipulation by parasites [24–27]. Parasites that target the neuronal system of the host alter the synthesis and concentration of neuromodulators like biogenic amine [28,29]. Parasites that target the immunological mechanism modulate the host's immune system, which acts on the host's nervous system and behaviour [30]. Altering the genomic and proteomic function of the host is another strategy used by the parasites to modify the host's behaviour [31]. Some parasites use their symbionts to prompt host manipulation [32]. However, in the case of direct bodyguard manipulation, the exact mechanism of manipulation is still unclear.

## 4. Bodyguard manipulation or stress-induced sleep?

The behaviour of the bodyguard-manipulated host resembles the moult-sleep behaviour of the caterpillar [33]. During moult sleep, *Ma. sexta* exhibits prolonged inactivity preceding the ecdysis. The larvae undergoing moult-sleep do not feed or move till ecdysis [34]. It is suggested that uncoupling of the feeding homeostat may be a reason for the prolonged quiescence bout in food-deprived larva during moult-sleep [35]. MacWilliam *et al*. [35] reported that the larva in moult-sleep responds to noxious stimuli with defensive strikes. Several stress conditions like hypertonicity, noxious temperature (heat and cold), ultraviolet exposure and cellular damage can also induce a similar sleep-like state in animals [36,37]. *Drosophila melanogaster* and *Caenorhabditis elegans* exhibit stress-induced sleep when exposed to injury or stress [38]. Prompting such a quiescence state in the host by the parasitoid can produce a guarding host with minimal effort.

In invertebrates, stress-induced sleep is studied using *Ca. elegans* and *Dr. melanogaster* as model organisms [39]*.* Cessation of pharyngeal pumping and locomotion are two main behaviours associated with stress-induced sleep in *Ca. elegans* [40]. Stress-induced sleep and developmentally timed sleep (similar to moult sleep in insects) in *Ca. elegans* are regulated by a neuropeptide, Orcokinin. Orcokinin is a conserved neuropeptide of Ecdysozoans. Orcokinin in *Ca. elegans* is encoded by the *nlp-14* gene present in the ALA neuron and its paralogue *nlp-15* genes in the ALA and RIS neurons [41]. ALA is a nociceptive neuron; epidermal growth factor (EGF) elevation in ALA neurons can induce behavioural quiescence in *Ca. elegans*. Heat stress in *Ca. elegans* causes depolarization of ALA neurons and the release of Phe-Met-Arg-Phe (FMRF) amide-like neuropeptides, resulting in loss of feeding and locomotion [42]. Similarly, tissue damage by pore-forming proteins in *Ca. elegans* can also induce stress-induced sleep with behavioural phenotypes like feeding and locomotion suppression, reduced responsiveness to weak stimuli, but rapid reversibility in response to strong stimuli [36].

In *Dr. melanogaster*, FMRF amide and its receptor play a vital role in stress-induced sleep [38]. Stress owing to infection or injury can trigger an immune response and stress-induced sleep in insects. In insects, the main characteristics of sleep are the absence of food intake, consolidated period of immobility and increased arousal threshold [43,44]. It is proposed that stress-induced sleep during an immune challenge is an evolutionary mechanism to overcome the challenge and to enhance the immune response [45]. An injury or immune challenge can induce nuclear factor κB (NFκB) Relish-dependent gene expression in fat bodies. The expression of *(NFκB) Relish* in fat bodies can promote sleep in insects [45]. In *Dr. melanogaster*, different stress modalities can induce sleep using different molecular mechanisms. However, all these signalling mechanisms converge to FMRF amide and its receptor to promote stress-induced sleep in *Dr. melanogaster* [38].

Compared to an aseptic injury, the immune challenge induced during parasitoid egression is massive [6]. It is demonstrated that only the emergence of parasitoid juveniles from the host is sufficient to induce feeding and locomotion suppression in the host [46]. Like the stress-induced sleep behaviour in *Ca. elegans* and *Dr. melanogaster*, post-parasitoid-egressed *Ma. sexta* also exhibits cessation of feeding and locomotion and responds to external disturbances [46]. Post-parasitoid-egressed *Ma. sexta* has reduced respiration and metabolic rate similar to a sleeping *Dr. melanogaster* [47,48]. Moreover, immunohistochemical studies show accumulation of FMRF amide-like neuropeptides in the brain neurosecretory cells and the axon terminals of corpora cardiaca-corpora allata complex of post-parasitoid-egressed *Ma. sexta* [49]. These pieces of evidence underpin that the changes in the behaviour of post-parasitoid-egressed host are probably the side effect of immune/stress-induced sleep triggered by the emergence of parasitoid pre-pupa/e. The phenotypes of post-parasitoid egressed host, invertebrates in stress-induced sleep and invertebrates in normal sleep-like or quiescence state are compared in table 1.

**Table 1.** The phenotypes of post-parasitoid egressed host, invertebrates in stress-induced sleep and invertebrates in normal sleep-like or quiescence state.

Sl. no.	phenotypes of post-parasitoid egressed host	phenotypes of stress-induced sleep in invertebrates	phenotypes of normal sleep-like state or quiescence state in invertebrates
1.	feeding and locomotion halt [19,46]	feeding and locomotion halt [40]	feeding and locomotion halt [43,50]
2.	low metabolic and respiratory activity [51]	unknown	low metabolism [48] discontinuous gas exchange [52]
3.	accumulation of FMRF amide like neuropeptide in brain-CC-CA complex [49]	FMRF amide like neuropeptide and its receptor FR responsible is involved [38,42]	FMRF amide like neuropeptides is not involved [42]
4.	low arousal threshold [19,53]	high arousal threshold [36,50]	high arousal threshold [43]
5.	high octopamine concentration [46,54]	unknown	low octopamine concentration [43]
6.	unknown	unknown	sleep rebound after sleep deprivation [43]
7.	unknown	unknown	stereotypic posture [43]

## 5. Immune and stress response during parasitoid emergence

In parasitized hosts, the emergence of the parasitoid juvenile activates a cytokine storm which causes a change in gene expression of various cytokines and antimicrobial peptides (AMP) like plasmatocyte spreading peptide (PSP), Attacin-1 and spätzle [6]. The inflammatory mediators and the AMP can regulate neuronal functions like neurotransmitter release, memory formation and sleep in insects [55–57]. Transcript levels of AMPs like *Metchnikowin (Mtk), drosocin (dro)* and *Attacin (Att)* increase respectively in glia, neurons and the head fat body during sleep deprivation [58]. After sleep deprivation, an antimicrobial peptide NEMURI (nur) is overexpressed in adult flies' heads. The *nur* RNA is also overexpressed during infection, which causes increased sleep during the period [59]. However, whether similar changes occur in gene expression of the parasitized host is yet to be studied.

Parasitoid emergence from the host's body also elevates octopamine concentration in the host haemolymph [46,54]. Octopamine is an analogue of vertebrate noradrenaline and a neurohormonal mediator of stress response in insects. The release of stress hormones during the immune challenge is common across the animal kingdom. The immune response is an energy-consuming process like a flight-and-fight response. Elevated octopamine concentration can increase lipid concentration of haemolymph and thus release a large amount of energy for the immune response [60]. The high octopamine concentration also shifts the carbohydrate-based energy metabolism to fatty acid-based energy metabolism, which involves releasing considerable energy [61].

Octopamine is also generally considered part of the arousal system in insects [62]. However, even with high levels of octopamine, the post-parasitoid-egressed host has a low level of arousal or a decreased tendency to initiate motor activities [46]. Increased octopamine in insects usually increases the locomotive activity but not in the case of post-parasitoid-egressed host [63]; but the removal of the supra oesophageal ganglion from the post-parasitoid-egressed host causes hyperkinesis, which indicates that loss of locomotive activity is owing to neural inhibition [64]. Similarly, the injection of octopamine or PSP proteins can suppress feeding behaviour in the unparasitized host [6,46]. It is likely that the high concentration of both octopamine and PSP proteins mimics the host's immune or stress response and eventually shifts it to a quiescence state. In an unparasitized host, feeding suppression owing to octopamine injection can be reversed by injecting octopamine antagonist phentolamine. However, in a post-parasitoid-egressed host, this relationship is decoupled owing to unknown reasons and cannot be regained by the phentolamine application [46]. One likely explanation for these phenomena can be the accumulation of an FMRF amide-like neuropeptide in the cerebral neurosecretory system of the post-parasitoid-egressed host [49]. The FMRF amide-like neuropeptide also accumulates in the gut, the nervous and endocrine system of the post-parasitoid-egressed host [65]. In *Ca. elegans,* overexpression of *flp-13*, the gene which encodes for the FMRF amide-like neuropeptide, is enough to induce a quiescence state even during active periods [42]. The relatively elevated amount of this neuropeptide in the post-parasitoid-egressed host compared to the experimentally starved *Ma. sexta* may be owing to the immune/stress response of parasitoid egression. Another explanation for the loss of feeding is that increased octopamine concentration can suppress the foregut peristalsis [46,66] and increase the sugar level of the host's haemolymph [67]. Increased sugar concentration can also inhibit feeding through a negative feedback loop.

## 6. Defensive response during parasitoid-induced sleep

Unlike normal sleep, hosts exhibit a low sensory threshold to the mechanical stimulus after parasitoid egression, making them an ideal bodyguard. They respond aggressively via defensive strikes, even to light brush strokes [19,53]. The caterpillar in the moult sleep state also exhibits defensive strikes, but the response is obtained against noxious stimuli like pinches on the dorsal horn or in contact with a hot soldering iron [35]. So, this may evoke a question, what makes the post-parasitoid-egressed host more sensitive?

Octopamine is known for increasing the sensitivity of some mechanosensory neurons [68]. The mechanosensory neurons in the leg of the spider, *Cupiennius salei,* increase its sensitivity to octopamine application [69]. The activity of the chordotonal organ, a type I mechanoreceptor in insects, increase its firing in the presence of octopamine [70]. In caterpillars, the hairs or setae present on their bodies act as mechanoreceptors [71]. Since the emergence of parasitoids increases the octopamine concentration, it might also increase the sensitivity of mechanoreceptors. The increased sensitivity may prompt the host to respond violently to minor disturbances to its mechanoreceptors. The parasitoid juvenile may use this opportunity by pupating on the dorsal or lateral side of the host body and thus have a perfect bodyguard from predators and hyperparasitoids [16,19].

## 7. Why does the host not recover from its stress-induced sleep?

Even if the parasitoid egression induces the quiescence state in the host, there remains a question of what keeps them in a prolonged sleep. *Manduca sexta* continues in its quiescence state for two weeks after the parasitoid egression and eventually dies in that state [72]. However, in the case of *Polysphincta gutfreundi,* an ichneumonid wasp that induces orb-weaving behaviour in the spider, the experimental removal of parasitoid larva results in a gradual recovery of the host to normal behaviour in two weeks [22]. The authors hypothesized that the spider's recovery happens dose-dependently based on the removal of parasitoid-derived substances that impact the host's nervous system [22]. In the case of *Col. maculata* parasitized by *Di. coccinellae,* some hosts recover from their bodyguard state within a week of adult parasitoid emergence and regain their normal feeding and reproductive behaviour [32].

In the recovered *Col. maculata*, the clearance of symbiotic virus, *Dinocampus coccinellae paralysis virus (DcPV),* occurs in their brains. The *DcPV* virus replicates inside the parasitoid larva and transmits to the ladybird host before the parasitoid egresses. The viral particles are found in the glial cells of the host before the parasitoid egresses. After the parasitoid egression, the glial cell appears vacuolated and shows signs of neuron degradation [32]. In vertebrates and invertebrates, glial cells regulate the sleep-like state. It was shown that the cultured glial cells secrete several molecules, which, when injected, can also increase sleep time in animals [73]. Glial-ablated animals exhibit prolonged sleep, locomotion quiescence and delayed development [74]. Unlike non-recovered hosts, the recovered *Col. maculata* host has limited neuron degradation and glial vacuolization, which explains their recovery from the quiescence state [32].

Like *Di. coccinellae,* bodyguard manipulative Microgastrinae parasitoids also transmit their symbiotic virus, polydnavirus (PDV), into the host body. However, unlike DcPV, an RNA virus, PDV is a DNA virus and is transmitted to the host during oviposition [75]. The viral particles transmitted into the host lack their replication machinery and hence cannot replicate inside the host body. Yet, PDV can integrate into the host genome using the host integrase enzymes [76]. Host-integrated PDV can suppress the host immune mechanism and alter the host physiology [77–82]. PDV can also modulate the host endocrine system and arrest the host metamorphosis. PDV blocks the phosphorylation of the target of rapamycin (TOR) pathway of the prothoracic gland despite being stimulated by prothoracicotropic hormone and thus inhibits the ecdysteroidogenesis of the host [83]. PDV is found to be integrated into the nervous system of the host even after six days of parasitoid egression, which opens the possibility of their role in neuronal protein regulation [84]. PDV also has an impact on the expression of neuropeptide in the host brain [85]. PDV injected into the unparasitized host can regulate the expression of pro-neuropeptide genes like FMRF amide and tachykinin in the host brain-corpora cardiaca-corpora allata complex [85]. These neuropeptides have a role in regulating gut muscle contraction. However, the injection of PDV into the unparasitized host does not evoke the quiescence state similar to that of the post-parasitoid-egressed host [6]. Also, there is no evidence that PDV causes any neuroinflammation and glial vacuolization or keeps the host in a quiscence state.

## 8. Conclusion and future perspective

**Figure 1.**
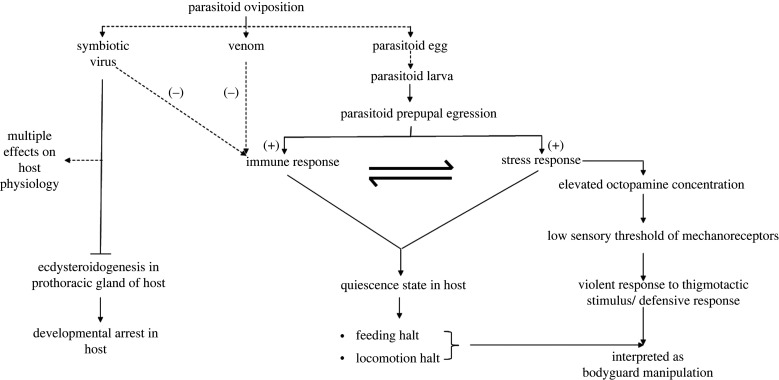
A possible model of direct bodyguard manipulation mechanism. The solid lines indicate the changes in the 5th instar host, while the dotted lines indicate the stages before the 5th instar.

Parasitoid–host interactions are dynamic and evolving rapidly. Parasitoids deploy different strategies for successful parasitization and protecting their offspring from hyperparasitoids. Among them, bodyguard manipulation is considered the most advanced strategy. Although a handful of studies have unravelled the cases of bodyguard-manipulating parasitoids and hosts, the fundamental mechanism of the process is a mystery. This review proposes a possible model of how the parasitoids might induce direct bodyguard manipulation, which is summarized in figure 1. Yet, it is challenging to offer a model applicable to all the direct bodyguard manipulation-inducing parasitoids. In our proposed model, we particularly addressed the proximate mechanism of behavioural changes induced by Microgastrinae parasitoids because most case studies of direct bodyguard manipulation are induced by Microgastrinae wasps. Also, the physiological changes induced by polydnavirus in Microgastrinae parasitoids (*Cotesia*, *Glyptapanteles and Microplitis sp.*) might differ from those caused by symbiotic RNA viruses associated with Helconoid wasps (*Dinocampus* sp.).

There are many lacunae in the knowledge of the mechanism behind host manipulation. Previous studies have concentrated on the host's immunological, neuronal and symbiont-mediated changes during manipulation. It should be combined with the advances in functional genomics, proteomics and histological analyses to elucidate the mechanism of behaviour in the manipulated hosts. This can further gain insights into the processes underlying the parasitoid–host interactions and the evolutionary patterns that they generate.

## Data Availability

Data are provided in the electronic supplementary material [86].
